# Analysis of the Toxicity and Histopathology Induced by the Oral Administration of *Pseudanabaena galeata* and *Geitlerinema splendidum* (Cyanobacteria) Extracts to Mice

**DOI:** 10.3390/md12010508

**Published:** 2014-01-22

**Authors:** Marisa Rangel, Joyce C. G. Martins, Angélica Nunes Garcia, Geanne A. A. Conserva, Adriana Costa-Neves, Célia Leite Sant’Anna, Luciana Retz de Carvalho

**Affiliations:** 1 Immunopathology Laboratory, Butantan Institute, Av. Vital Brasil, 1500, Sao Paulo SP 05503-900, Brazil; E-Mail: joycecarolmartins@ig.com.br; 2 Phycology Section, Institute of Botany, Av. Miguel Stéfano, 3687, Sao Paulo SP 04301-902, Brazil; E-Mails: angelsgarcia@uol.com.br (A.N.G.); geanne.conserva@yahoo.com.br (G.A.A.C.); celialsant@yahoo.com.br (C.L.S.); lretz@uol.com.br (L.R.C.); 3 Department of Genetics, Butantan Institute, Av. Vital Brasil, 1500, Sao Paulo SP 05503-900, Brazil; E-Mail: adriana.neves@butantan.gov.br

**Keywords:** water reservoirs, cyanobacteria, cyanotoxins, mouse toxicity, histopathology

## Abstract

Cyanobacteria are common members of the freshwater microbiota in lakes and drinking water reservoirs, and are responsible for several cases of human intoxications in Brazil. *Pseudanabaena galeata* and *Geitlerinema splendidum* are examples of the toxic species that are very frequently found in reservoirs in Sao Paulo, which is the most densely populated area in Brazil. In the search for toxic strains collected from water reservoirs and maintained in the Cyanobacterial Culture Collection (CCIBt) of the Institute of Botany of Brazil, the acetic acid extracts (AE) of *P. galeata* CCIBt 3082 and *G. splendidum* CCIBt 3223 were analyzed by planar chromatography, which indicated the absence of cyanotoxins. Animal tests were then carried out, and both extracts were found to induce toxic effects in mice when administered intraperitoneally. The present study aimed to investigate whether the oral ingestion of the above mentioned cyanobacteria extracts would also induce toxic effects in mice. Necropsy and histopathological studies were conducted using tissue samples from the animals, which were euthanized one week after the administration of the extracts. The AE of *P. galeata* did not cause death but did induce transient symptoms, including eyebrow ptosis, straub tail, and pain. The euthanized animals presented hemorrhage in the liver, whereas the histological analysis showed disorganization of the hepatic parenchyma, necrosis, hyperemia, and proximity of the centrilobular vein in the liver. In addition, alterations in the convoluted tubules of the kidneys were observed, and the lungs were unaffected. The AE of *G. splendidum* caused only one death, and induced transient symptoms, such as dyspnea, paralysis, and pain, in the other mice. The necropsy of the euthanized mice showed hemorrhage in the lungs and liver. The lungs presented hemorrhagic focuses, alveolar collapse, and granulomatous foci. The liver presented hemorrhagic and enlarged sinusoids, hyperemia, proximity of the centrilobular vein, and disorganization of the hepatic parenchyma. Some areas also exhibited an inflammatory infiltrate and calcified tissue inside blood vessels. Necrosis and rupture of the convoluted tubule cells were observed in the kidneys. Further analysis of the both extracts indicated the lack of hemolytic activity, and the presence of two unknown anti-AChE substances in the AE of *G. splendidum*. Thus, *P. galeata* and *G. splendidum* are producers of novel toxins that affect mammals when administered orally.

## 1. Introduction

Many bioactive natural products have been characterized from cyanobacteria in the past few years [1,2,3,4,5,6,7]. Some compounds are of medical and/or pharmaceutical importance as they exhibit antimicrobial, antifungal, and anticancer activities [8,9]. However, some of these metabolites are toxic toward a great variety of organisms, including humans [10,11].

According to the review compiled by Skulberg *et al*. [12], of the two thousand cyanobacteria species comprising 150 genera, 40 species are toxic. There is much to be investigated in this field of research as only 5%–10% of the cyanobacterial biodiversity from the tropical regions was evaluated in terms of their taxonomy and toxicity until the last decade [13]. Cyanobacteria toxins, which are also called cyanotoxins, can affect the gastric, hepatic, and neural systems, and can induce mutagenic and teratogenic effects, toxic effects on gonads and embryos, and tumor promotion [14]. Cyanotoxins are classified as hepatotoxins, neurotoxins, cytotoxins, dermatotoxins, and/or irritant toxins according to their effects on mammals [15,16,17].

A cyanobacterial bloom formation may occur in a water body under specific conditions. The release of cyanotoxins, such as microcystins, saxitoxins, anatoxin-a, anatoxin-a(*S*), and cylindrospermopsins, in water reservoirs that are used for human consumption can cause public health problems [16,18,19,20,21]. These substances can be the source of acute lethal intoxications or may induce the development of chronic diseases, e.g., the continuous ingestion of sub-lethal doses of microcystins promotes the growth of hepatic tumors or necrosis [22,23].

The current legislation in Brazil on cyanobacteria toxins in water reservoirs for human and animal consumption is mandatory only for microcystins and saxitoxins, whereas the analysis of the water for the presence of cylindrospermopsin and anatoxin-a(*S*) is only performed if cyanobacteria that potentially produce these compounds are observed [24].

In our studies of Brazilian cyanobacteria, we demonstrated that some species that do not typically form blooms but are frequently found in our water reservoirs and urban lakes produce toxins other than the known cyanotoxins. As a standard procedure of the screening program for toxic strains collected from water reservoirs and maintained in the Cyanobacterial Culture Collection (CCIBt) of the Institute of Botany of Brazil, the extracts of all cyanobacteria are analyzed by HPTLC (high performance thin layer chromatography) to detect cyanotoxins (microcystins, saxitoxins, anatoxin-a, and β-methylamino-l-alanine). Only the extracts with negative results (no cyanotoxin) are tested in mice bioassay. According to Harada *et al.* [25], mouse bioassay is the standard test for toxicity evaluation of biomass produced by blooms or cultures of dinoflagellates and cyanobacteria. This test is also important to characterize cyanobacteria toxins, as the symptoms presented by intoxicated animal, the time to death after injection, and post-mortem examination may indicate the toxin’s nature. Furthermore, bioassays are necessary when there is indication of the presence of cyanotoxins other than (or in addition to) the known toxicants [25]. Although cell-based assays (or ELISA kits or other analytical methods) for standard cyanotoxins detection are relevant to monitoring programs, they will not provide enough evidence that animals or people can be intoxicated or poisoned by ingesting the cyanobacteria or their cell’s contents present or released in the water reservoirs when the presence of unknown toxins is suspected. 

Two species from the *Geitlerinema* genus exhibited toxicity to mice (i.p.—intraperitoneal administration) due to the presence of pro-inflammatory and antiacetylcholinesterase (anti-AChE) substances in their extracts: the methanol extract (ME) of *Geitlerinema unigranulatum* CCIBt 3213 [17,26,27] and the acetic acid extract (AE) of *Geitlerinema splendidum* CCIBt 3223 [17,28]. *Geitlerinema* spp. does not usually form blooms, but the CCIBt 3223 strain was found in the Guarapiranga reservoir in Sao Paulo City, which is a eutrophic drinking water reservoir in the highest populated area of Brazil [17].

Another species that has been studied by our group is *Pseudanabaena galeata*. The strain *P. galeata* CCIBt 3082 was also collected in an urban lake in Sao Paulo. Its AE caused hepatic damage when administered intraperitoneally to mice, and body weight reduction and tumor promotion was observed in the liver one week after the administration of a single dose [29].

In the present study, we performed bioassays to determine the toxicity induced by the oral administration of the AEs of *P. galeata* CCIBt 3082 and *G. splendidum* CCIBt 3223 to mice. This study aimed to demonstrate that the ingestion of the aqueous content of these cyanobacteria can induce toxicity symptoms or death in mammal and affect the histological aspects of vital organs. We also investigated whether the toxins exert an unspecific effect in cell membranes through a hemolytic assay with mouse erythrocytes.

## 2. Results

**Figure 1:**
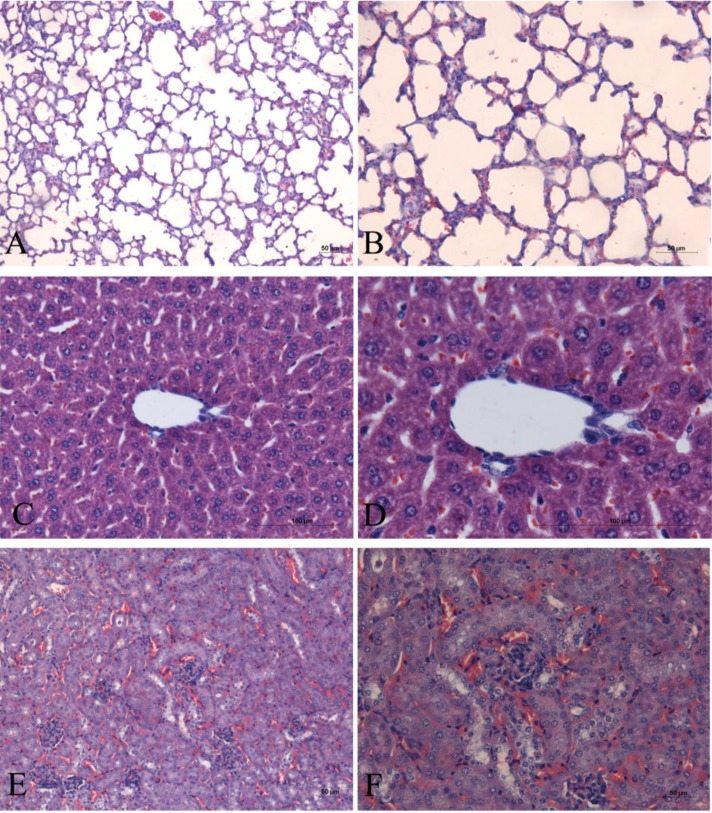
Histological sections of the organs of control mice stained with hematoxylin and eosin. (**A**,**B)** lung; (**C**,**D**) liver; (**E**,**F**) kidney.

The mice administered with Milli-Q exhibited no intoxication symptoms, presented no post-mortem alterations in the necropsy of the euthanized animals, and showed no microscopic alterations in the organs (lungs, kidneys, and liver), as shown in Figure 1.

**Figure 2:**
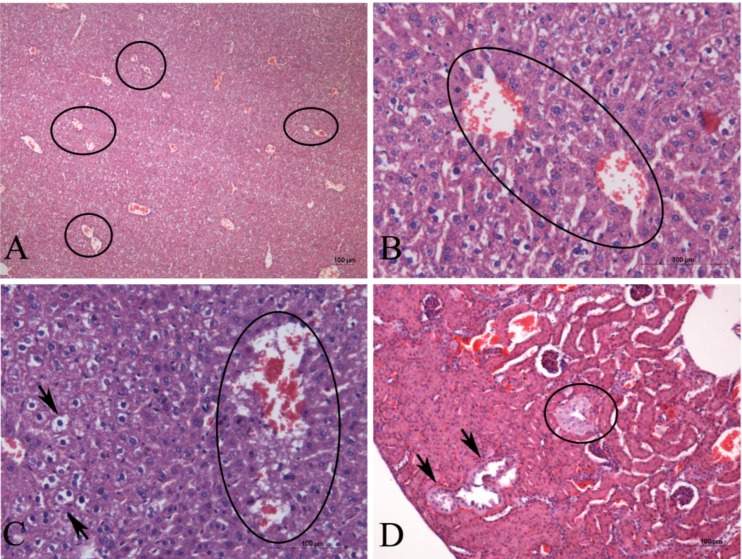
Histological sections of mice organs after the oral administration of the AE of *P. galeata* (1 g kg^−1^ b.w.). The liver is shown in **A**, **B**, and **C**, and the kidney is shown in **D**. (**A**) Hyperemia and proximity of the centrilobular vein (circles) and disorganization of the hepatic parenchyma. (**B**) Proximity of the centrilobular vein (circle) and disorganization of the hepatic parenchyma (circle). (**C**) Necrosis (circle), vacuolization of liver cells (arrows), and disorganization of the hepatic parenchyma. (**D**) Alterations in the convoluted tubules, characterized by cellular hypertrophy (circle) and expansion of the tubules (arrows).

At a dosage of 1 g kg^−1^, the AE *P. galeata* CCIBt3082 did not cause death but induced various symptoms, including eyebrow ptosis, straub tail, and pain. One week after administration, the euthanized animals presented hemorrhage in the liver. The histology showed disorganization of the hepatic parenchyma, necrosis, hyperemia, and proximity of the centrilobular vein (Figure 2A–C). In addition, the kidneys presented enlargement and cellular hypertrophy of convoluted tubules (Figure 2D), and the lungs were unaffected.

**Figure 3:**
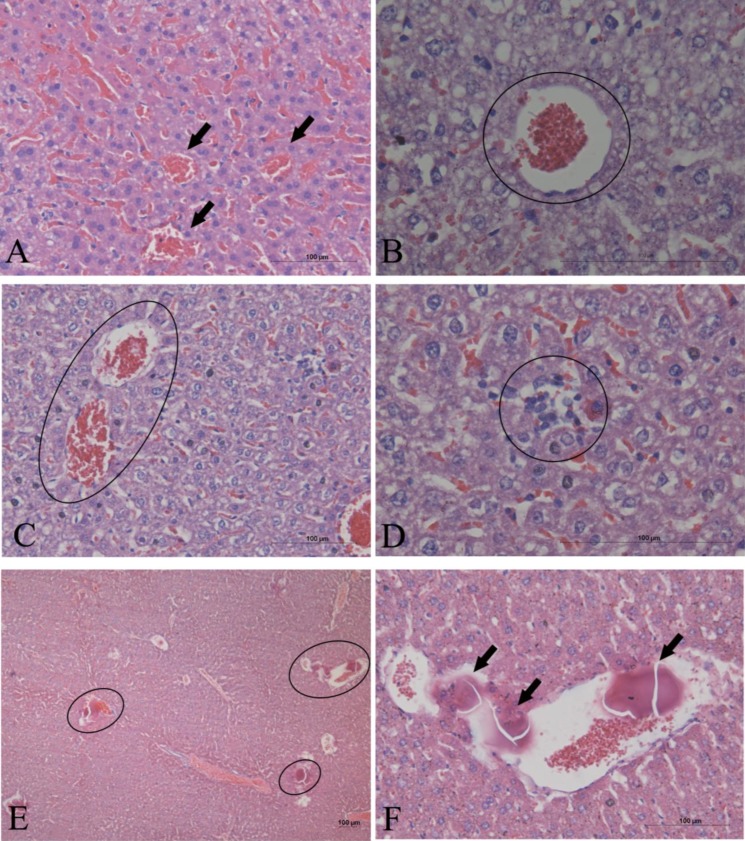
Histological sections of mice lungs after the oral administration of the AE of *G. splendidum*. The sections were stained with hematoxylin and eosin, (**A**,**B**) 0.5 g kg^−1^ body weight (b.w.); hemorrhagic focuses (circle), artery distention (square), and alveolar collapse. (**C**,**D**) 1 g kg^−1^ b.w.; hemorrhagic focuses (circle). (**E**,**F**) 2 g kg^−1^ b.w.; hemorrhagic focuses (square) and granulomatous foci (circle).

**Figure 4:**
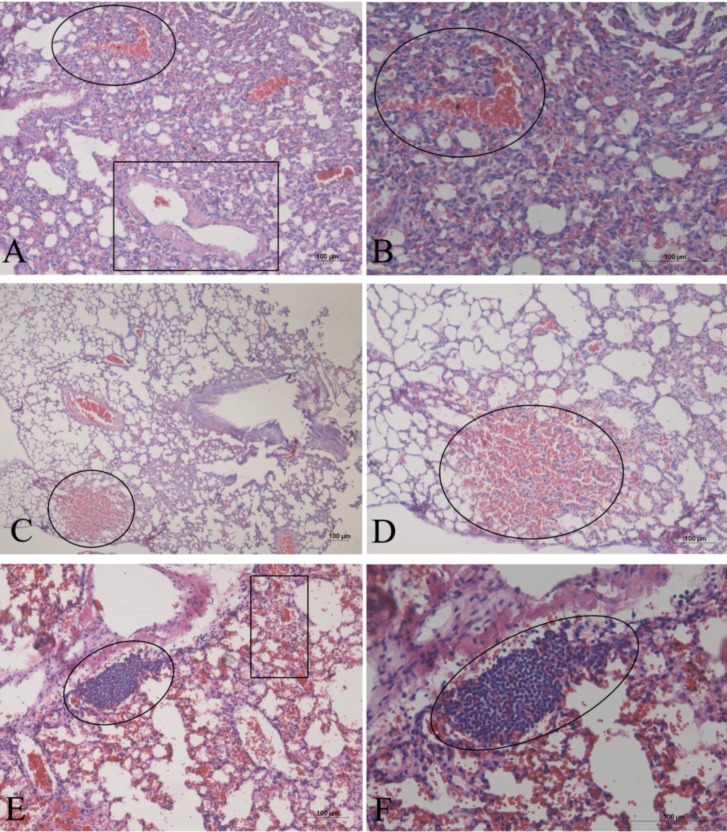
Histological sections of mice liver after the oral administration of the AE of *G. splendidum*. The sections were stained with hematoxylin and eosin. (**A**,**B**) 0.5 g kg^−1^ b.w.; hemorrhagic and enlarged sinusoids (arrows) and hyperemia of centrilobular vein (circle). (**C**) 1 g kg^−1^ b.w.; hyperemia and proximity of the centrilobular vein (circle). (**D**) 1 g kg^−1^ b.w.; inflammatory infiltrate (circle) and disorganization of the hepatic parenchyma. (**E**,**F)** 2 g kg^−1^ b.w.; same observations shown in A-D and calcified tissue inside blood vessels (circles and arrows).

**Figure 5:**
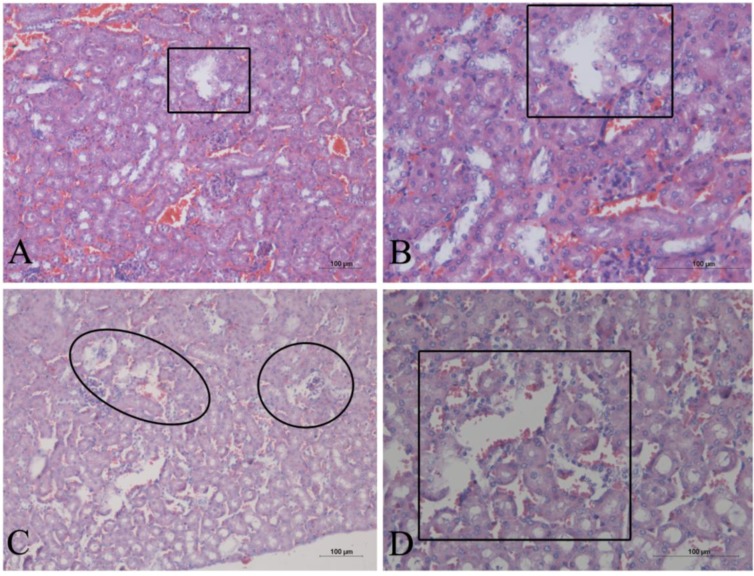
Histological sections of mice kidneys after the oral administration of the AE of *G. splendidum*. The sections were stained with hematoxylin and eosin. (**A**,**B**) 0.5 g kg^−1^ b.w.; necrotic area in the convoluted tubules (square). (**C**,**D**) 1 g kg^−1^ b.w.; necrosis (circle) and rupture of the convoluted tubules (square).

At dosages of 0.5, 1, and 2 g kg^−1^, the AE *G. splendidum* CCIBt 3223 induced dyspnea, paralysis, and pain (and one death at a dosage of 0.5 g, kg^−1^), which indicates that this strain presents low toxicity according to the scale described by Lawton *et al*. [30]. The necropsy of the euthanized mice showed hemorrhage in the lungs and liver. The lungs presented hemorrhagic focuses, alveolar collapse, and granulomatous foci (macrophages) (Figure 3). The liver presented hemorrhagic and enlarged sinusoids, hyperemia, proximity of the centrilobular vein, and disorganization of the hepatic parenchyma (Figure 4A–D). Some areas presented inflammatory infiltrate (Figure 4D) and calcified tissue inside the blood vessels (Figure 4E–F). Necrosis and rupture of convoluted tubule cells were observed in the kidneys (Figure 5).

Even at high concentrations of 1 mg/mL of a 4% suspension of mouse erythrocytes (ES), the AEs of *P. galeata* CCIBt 3082 and *G. splendidum* CCIBt 3223 exhibited no hemolytic activity against murine red blood cells.

## 3. Discussion

Previous studies performed as a standard procedure of our screening program at Cyanobacterial Culture Collection of the Institute of Botany of Brazil involved HPTLC experiments to detect the presence of microcystins, saxitoxins, anatoxin-a, and BMAA (accordingly to methods described by [31,32,33,34]). The AEs of *P. galeata* CCIBt 3082 and *G. splendidum* CCIBt 3223 presented negative results [28,35]. As both strains are very frequent in our water reservoirs in Sao Paulo area, those where considered as of possible toxicological importance and mice toxicity experiments were conducted to detected the presence of other toxins.

Acute and chronic mice intoxication symptoms and histopathological effects of the most common cyanotoxins have already been described thoroughly in the literature. The signs that indicate the presence of alkaloid neurotoxic cyanotoxins, such as saxitoxins and anatoxins, are: muscle progressive paralysis, overrated abdominal breath, cyanosis, convulsions, respiratory arrest, and death in a few minutes after sample administration, and absence of organ alterations in the post-mortem exam [25]. Differently, the protein phosphatases inhibitors microcystins and nodularins, cyanobacteria hepatotoxic peptides, induce diarrhea, vomit, piloerection, weakness, and paleness in the intoxicated animals. Death occurs from 50 min to 2 h after administration, and the autopsy shows augmented and hemorrhagic liver. Lethal doses of microcystins also induce pulmonary inflammation, congestion, and hemorrhage [36]. Sublethal chronic administration of microcystins causes hepatocytomegaly and karyomegaly with moderate parenchymal disarray in mice liver after four to seven daily doses. Multinucleated hepatocytes containing as many as seven nuclei per cell were commonly seen, mostly adjacent to the central vein, and cytoplasmic vacuolation and apoptosis were also present [37]. Cylindrospermopsin is a toxin that inhibits the protein synthesis in the cells (*in vitro* and *in vivo*). When administrated in mice reduces the levels of mobility and responsiveness, causes mild piloerection and diarrhea. The deaths occur at least after 6 h after i.p. administration up to six to seven days depending on the dose [38,39,40]. Oral or intraperitoneal administration of cylindrospermopsin in mice induces, after 24 h, primary DNA damage in the colon as well as cell death in the liver and kidneys [41]. The lipopolysacharides (LPS), cell wall components of gram-negative bacteria, are pyrogenic (fever causing agents) and toxic. The few studies carried out on cyanobacterial LPS indicate that they are less toxic than the LPS of other bacteria, such as *Salmonella*. Furthermore, acute LD_50_ values in mice are at least tenfold higher than the doses used at the present work, in addition, the method for extraction of cyanobacterial LPS is different than ours ([39,42] and cited references).

The AEs of *P. galeata* CCIBt 3082 and *G. splendidum* CCIBt 3223 are toxic to mice when injected intraperitoneally and induce microscopic lesions in vital organs, as previously described [17,29]. We conducted this study to determine whether the observed toxic effects after intraperitoneal administration also occur when the same extracts are administered orally.

The literature on the toxic effects of *P. galeata* is scarce. Teneva *et al*. [43] verified the presence of hepatic and kidney lesions in mice after the intraperitoneal administration of the extract. No mice died after the extract administration, and the liver histology showed granulovacuolar degeneration and mitosis, inflammatory cellular infiltration, obscured cell borders, congestion, hemorrhage, and necrosis, five days after the administration of the extract. The histological alterations in the renal tissue of the treated mice included hemorrhage, inflammation between tubules, necrosis and destruction of tubular cells, and atrophy of the glomerulus. Microcystins, saxitoxins, and anatoxin-A were detected in the *P. galeata* extract by HPLC and ELISA [43]. Microcystin is a hepatotoxin, whereas the other two toxins are considered neurotoxins with a rapid effect. In our experiments, we also verified the presence of liver and kidney lesions in mice after the oral administration of the AE of *P. galeata* CCIBt 3082 (Figure 2). However, the intoxication symptoms presented (eyebrow ptosis, straub tail, and pain) and several of the developed lesions observed through histological analysis (hyperemia, proximity of the centrilobular vein, necrosis in the liver, and enlargement and cellular hypertrophy of convoluted tubules in the kidney) were different from those described by Teneva *et al*. [43] and different from the signals typically described for microcystin intoxication [25,36,37], as explained above. The pain symptoms observed in the mice in our experiment may have been caused by the necrosis observed in the liver because pain due to widespread necrosis in a range of tissues would likely have resulted in hind region effects [44].

The oral administration of the AE of *G. splendidum* CCIBt 3223 generally induced the same symptoms and lesions in mice that were induced by its intraperitoneal administration [17]. The observed intoxication symptoms did not resemble the effects provoked by hepatotoxins, such as microcystins, but hepatic hemorrhage was visible at the post-mortem exam. Neurotoxins (such as saxitoxin and anatoxin), LPS, and cylindrospermopsins would also cause different symptoms, or were absent in the extract due to the extraction methods employed in this work. Furthermore, the chemical analysis of this extract indicated the absence of both microcystin and saxitoxin [28,35].

The subsequent histological analysis of the liver, lungs, and kidneys corroborated the hypothesis that a different toxin is present in these extracts because the analysis of the tissues and cells, particularly the lungs, indicated the presence of a pro-inflammatory substance. The augmented number of cells in the lung tissue must be related to the recruitment of inflammatory cells, such as macrophages. The extracts of another *Geitlerinema* species also exhibited pro-inflammatory activity [26]. Another interesting observation was the evidence of healing processes in the liver, as suggested by the proximity of the centrilobular veins and disorganization of the hepatic parenchyma. This finding was most likely observed because the animals were euthanized eight days after the administration of the extract; thus, it is possible that the organs lesions started to heal. Similar cellular damage and tissue repair in the mice liver was observed after the intraperitoneal administration of a *Limnothrix* sp. extract. The infiltration of leukocytes, macrophages, and neutrophils was observed in the liver, kidneys, and lungs. Furthermore, this *Limnothrix* sp. strain exhibited morphological characteristics that were very similar to those of *Geitlerinema unigranulatum* [44].

More recently, two substances with antiacetylcholinesterase (anti-AChE) activity were detected by TLC autography in the AE of *G. splendidum* CCIBt 3223 [28]. When injected into mice (i.p.), the extract containing these anti-AChE substances indicated a lethal anti-AChE activity, most likely by the presence of long acting (irreversible and, thus, toxic) inhibitory substances to the enzyme. When administrated *per os* the absorption of toxic components can be reduced, therefore in the results presented here only one death occurred (at the dosage of 0.5 g kg^−1^) and the symptoms where mild when compared to i.p. administration. Two symptoms observed in the mice following the oral administration of the AE of *G. splendidum* CCIBt 3223 could be associated with the systemic effects induced by an anti-AChE drug: dyspnea, which is related to the muscarinic receptors, while paralysis is related to the overstimulation of the nicotinic ACh receptors [28].

Anti-AChE substances have also been identified in a Brazilian strain of *G. unigranulatum* CCIBt 3213 [27] and were found to cause intoxication symptoms and death when administered intraperitoneally to mice.

In addition, neither the AE of *P. galeata* CCIBt 3082 nor the AE of *G. splendidum* CCIBt 3223 induced hemolysis in murine erythrocytes at concentrations up to 1 mg/ml 4% ES. This experiment was performed because the unspecific effects on the cell membrane caused by hemolytic substances may be responsible for mouse death, neurotoxic symptoms, respiratory arrest, pain, and tissue damage [45]. Some previous studies indicate that substances that are haemolytic or cytotoxic due to pore formation in membranes can also present neurotoxic properties [45,46,47,48,49,50,51,52,53,54]. Pore insertion may occur into the cell membranes of various target organs. Hemolysis can, thus, be explained on the basis of an osmotic misbalance of the red cells as consequence of pore induction. Neurotoxic effects, on the other hand, result most probably from widespread nerve membrane depolarization caused by the shunting action of the induced pores. These toxins generally form ion channels that allow the inward flow of Ca^2+^ and other ions to the pre-synaptic terminals, increasing neurotransmitter release at the neuromuscular junction, and the quanta content of the evoked endplate potentials, producing a progressive membrane depolarization [45]. Membrane depolarization of excitable cells will prevent further action potential conduction and post-synaptic excitability, causing paralysis, and ultimately death by respiratory failure.

## 4. Experimental Section

### 4.1. Biomass Production

The strains are maintained in liquid media (ASM-1 and BG-11) under controlled conditions: temperature, 23 ± 1 °C; irradiance, 40–50 µmol m^−2^ s^−1^; and photoperiod, 14-h light/10-h dark [55]. The biomass was produced by cultivating the unialgal nonaxenic strain in 9 L of ASM-1 under the same illumination and temperature conditions described above with constant stirring (70 rpm) or aeration until the exponential growth phase was reached. The cells were harvested by centrifugation and frozen at −20 °C.

### 4.2. Preparation of Cyanobacteria Extracts

For each strain, the freeze-dried cells were extracted with 0.1 M (4×) aqueous acetic acid or a 75:25 (v/v) methanol/water solution by sonication (4×, 10 s, and 50 W). The combined extracts were centrifuged (1830 × *g*, 50 min), and the supernatants were freeze dried and stored at −18 °C [56].

### 4.3. Mouse Bioassay

The use of mice in our experiments was in agreement with the Ethical Principles in Animal Research that were adopted by the Brazilian College of Animal Experimentation and was approved by the Ethical Committee for Animal Research of Butantan Institute (Protocol No. 385/07). The toxicity tests were performed in triplicate, and Swiss-Webster male mice (weighing 19–21 g) were used according to the method recommended by the World Health Organization [25], which consists of the administration of a cyanobacteria lysate. The mice were observed for 24 h and then sacrificed using an approved method [10].

Each mouse was orally administered a single dose of 0.5, 1, or 2 g of dried cells per kg of b.w. (body weight) in 0.2 mL of Milli-Q water, which enabled the extracts to be ranked as low-toxicity extracts if an animal death was caused [30]. The symptoms of intoxication and time to death were observed up to the 8th day after the administration of the extract. According to Harada *et al*. [25], the observation period must be extended to seven days when cylindrospermopsin is suspected. Although we did not particularly suspect of the presence of cylindrospermopsin, we extended the observation period, as was previously performed in the intraperitoneal toxicity assay of *P. galeata* [29] and *Geitlerinema* spp. [17] extracts. In both studies, the results indicated that the tissue injury assessment one week after administration is very relevant to this type of investigation. Prolonged observation periods have also been used by other researchers in studies of new groups of cyanobacteria and potentially novel toxins [43,44]. The control animals received 0.2 mL of Milli-Q through gavage. The mice that survived until the end of the observation period were euthanized with CO_2_. Post-mortem exams were performed in all of the animals to macroscopically observe the appearance of the vital organs (liver, heart, lungs, kidneys, and intestine) and to collect tissue samples for histology (liver, lungs, and kidneys).

### 4.4. Histological Analysis

The liver, lung, and kidney samples collected from the mouse bioassays were fixed in 10% buffered formalin, processed through conventional histological techniques, and stained with hematoxylin and eosin. Microscopy was performed using an optical microscope (Olympus BX51) equipped with a camera (Olympus Q-Color-5), and the images were recorded in a computer using the Image Pro-Express software.

### 4.5. Hemolytic Assay

To investigate the possible role of hemolysins, phospholipases, and other toxins that may affect the cell membranes as a cause of the murine toxicity, intoxication symptoms, or lesions in the organs, an hemolytic assay was performed. A 4% suspension of mouse erythrocytes (ES) was prepared as previously described [57,58]. Different concentrations of the extracts were incubated with the ES at room temperature (±22 °C) in an ELISA plate (96 wells) for 1 h and centrifuged (1085 × *g* for 5 min). The hemolytic activity of the supernatant was measured by the absorbance at 540 nm using the absorbance of the Krebs-Henseleit physiological solution (in mM: NaCl, 113; KH_2_PO_4_, 1.2; KCl, 4; MgSO_4_, 1.2; CaCl_2_, 2.5; NaHCO_3_, 25; and glucose, 11.1), which was the vehicle used to administer the extracts, as a blank. Total hemolysis was obtained with 1% Triton X-100, and the percentage of hemolysis was calculated relative to this value.

## 5. Conclusions

Together with the results previously published by our group [17,28,29], the results of the present study clearly indicate that *P. galeata* CCIBt 3082 and *G. splendidum* CCIBt 3223 are producers of novel water-soluble toxins that affect mammals when administered orally. Both species studied are often found in freshwater habitats, including drinking water reservoirs in a highly populated area, raising the question if small amounts of non-monitored toxins can be ingested by the local population. Thus, further work is necessary to chemically and pharmacologically characterize these toxins.
